# Herbicide bioremediation: from strains to bacterial communities

**DOI:** 10.1016/j.heliyon.2020.e05767

**Published:** 2020-12-24

**Authors:** Marcos Pileggi, Sônia A.V. Pileggi, Michael J. Sadowsky

**Affiliations:** a Laboratory of Environmental Microbiology, Biological Science and Health Institute, Department of Structural and Molecular Biology, and Genetics, State University of Ponta Grossa, Ponta Grossa, Paraná, Brazil; b The Biotechnology Institute, Department of Soil, Water, and Climate, Department of Plant and Microbial Biology, University of Minnesota, Saint Paul, MN, USA

**Keywords:** Environmental microbiology, Analytical chemistry, DNA analyses, Metabolic pathways, Cell communication

## Abstract

There is high demand for herbicides based on the necessity to increase crop production to satisfy world-wide demands. Nevertheless, there are negative impacts of herbicide use, manifesting as selection for resistant weeds, production of toxic metabolites from partial degradation of herbicides, changes in soil microbial communities and biogeochemical cycles, alterations in plant nutrition and soil fertility, and persistent environmental contamination. Some herbicides damage non-target microorganisms via directed interference with host metabolism and via oxidative stress mechanisms. For these reasons, it is necessary to identify sustainable, efficient methods to mitigate these environmental liabilities. Before the degradation process can be initiated by microbial enzymes and metabolic pathways, microorganisms need to tolerate the oxidative stresses caused by the herbicides themselves. This can be achieved via a complex system of enzymatic and non-enzymatic antioxidative stress systems. Many of these response systems are not herbicide specific, but rather triggered by a variety of substances. Collectively, these nonspecific response systems enhance the survival and fitness potential of microorganisms. Biodegradation studies and remediation approaches have relied on individually selected strains to effectively remediate herbicides in the environment. Nevertheless, it has been shown that microbial communication systems that modulate social relationships and metabolic pathways inside biofilm structures among microorganisms are complex; therefore, use of isolated strains for xenobiotic degradation needs to be enhanced using a community-based approach with biodegradation pathway integration. Bioremediation efforts can use omics-based technologies to gain a deeper understanding of the molecular complexes of bacterial communities to achieve to more efficient elimination of xenobiotics.

With this knowledge, the possibility of altering microbial communities is increased to improve the potential for bioremediation without causing other environmental impacts not anticipated by simpler approaches. The understanding of microbial community dynamics in free-living microbiota and those present in complex communities and in biofilms is paramount to achieving these objectives. It is also essential that non-developed countries, which are major food producers and consumers of pesticides, have access to these techniques to achieve sustainable production, without causing impacts through unknown side effects.

## 1 Introduction

Agriculture is constantly trying to increase of productivity. One strategy to achieve this goal is the use of herbicides. These chemical agents act by blocking the biosynthesis of amino acids, carotenoids or lipids, or by interrupting the flow of electrons in the process of photosynthesis. Nevertheless, massive use of herbicides and other pesticides leads to contamination of agricultural soils, river systems, and nearby groundwater, changing the structure and function of soil microbial communities. Herbicides directly or indirectly impact organisms other than their primary targets, including human. Herbicide use and misuse also causes selection pressure on microbes in soil and water, possibly resulting in changes to microbial processes, especially if there are genes encoding enzymes related to herbicide degradation. Finally, xenobiotic compounds may increase the production of reactive oxygen species (ROS). These compounds affect the survival of microorganisms that subsequently need to develop strategies to adapt to these conditions to maintain their ecological functionality. Without adaptation, specific populations of microorganisms will likely disappear.

Researchers have tested the use of several bioremediation technologies, aiming to render environmental herbicide contaminants non-harmful or less harmful. These promising environmental technologies are based on microbial metabolic activities (via enzymes) to transform toxic components into harmless molecules. While some microorganisms able to transform recalcitrant compounds have indeed been isolated, trials of their use in environmental applications have been disappointing.

Therefore, before degradation is achieved, microbial communities must develop survival strategies against the stresses induced by herbicides. Adaptation of bacteria to stressful environments is achieved by the interaction of several systems in a complex manner. A better understanding of these interactions has been achieved via novel molecular investigations of microbiota using various omics-based studies, all aiming to improve understanding of the complex relationships that constitute the systems of bacterial responses to resistance and the capacity for degradation of herbicides. There are consortia of various species with complex integrated networks of degradation mechanisms that function via coordinated quorum sensing systems. Currently, these can only be fully dissected and exploited using omics-based technologies. Moreover, there remains concerns that agricultural countries with high levels of contamination by pesticides and lesser degrees of scientific development will not be able to use these methodologies to maintain a broad level of ecological sustainability.

## 2 The importance of herbicides for agriculture

Commercial farmers are constantly trying to increase food crop production to satisfy worldwide demands. The World Health Organization data from 2016 (http://www.fao.org/sustainable-development-goals/indicators/211/en/) indicate that 11% of people in the world are undernourished, concentrated in African and Asian countries. One strategy to increase food crop production involves use of herbicides. These chemicals act by blocking the biosynthesis of amino acids, carotenoids, lipids, or interrupting the flow of electrons in the process of photosynthesis in weeds.

## 3 Herbicide modes of action

The rapid development of new tools for synthetic organic chemistry during the late 20^th^ century led to the synthesis of a variety of useful compounds. Among these were synthetic, herbicides, fungicides, insecticides, rodenticides, nematicides, and plant growth-promoting compounds (Aktar et al., 2009) that collectively improved crop production and reduced the weed-related damage. Some of these compounds target light harvesting or photosynthesis reactions, cell metabolism, or growth/cell division processes in weed plants (Table 1, Supplementary Material).

Herbicides have been classified into 25 groups (Beffa et al., 2019, www.hracglobal.com) based on inhibited proteins, targets, or via the similarity of induced symptoms [Herbicide Resistance Action Committee (HRAC) Herbicide Classification System]. In 2020, 261 herbicides were classified according to this system (www.hracglobal.com). A closer look at the chemical structures of these herbicides reveals a common factor: many have residues with high electronegativity that aid in the disruption of their targets or any other structure susceptible to oxido-reductive destruction, whether in weeds or non-target organisms. This review describes reactivities of some of the most commonly-cited herbicides in the literature, and widely used around the world (Maggi et al., 2019), for which there is more information regarding their degradation as well as the responses they induce in various organisms.

Some of these important herbicides and their modes of action are displayed in Table 1 (Supplementary Material). Other herbicides can be described according to this classification system. Imazethapyr is a chiral herbicide used in production of rice, soybeans, peanuts, and other crop plants. Imazethapyr is classified in the imidazolinone chemical family and the B HRAC group, affecting cell metabolism in their targets (Beffa et al., 2019). This herbicide selectively controls dicotyledonous weeds, inhibiting the synthesis of acetohydroxyacid or acetolactate. This enzyme catalyzes the initial reaction in the biosynthetic pathway for the branched chain amino acids valine, leucine, and isoleucine. Alachlor, acetochlor, butachlor, and metolachlor are chloroacetamides used against annual broad-leaved weeds. These chemicals are classified in the K3 HRAC group, affecting growth/cell division of their targets (Beffa et al., 2019).

Paraquat has high reducing potential and can capture electrons from photosystem I and decrease the concentration of NADPH^+^. The free electrons generated by this system are often reactive with the herbicide and can induce the formation of free radicals. These are converted to their original form by oxygen in a cycle that can harm various cell structures; eventually it may exhaust the supply free electrons for the continuation of the process (Gravina et al., 2017). The mode of action occurs via inhibition of protoporphyrinogen. This herbicide belongs to the bipyridylium chemical family and is classified in the D HRAC group, affecting light processes in their targets (Beffa et al., 2019).

Azimsulfuron is used for controlling weeds in paddy fields. This sulfonylurea herbicide inhibits the enzyme acetolactate synthase, which is involved in the biosynthesis of branched chain amino acids in plants and microorganisms. Azimsulfuron belongs to sulfonylurea chemical family and is classified in the B HRAC group, affecting the cell metabolism of their targets (Beffa et al., 2019).

## 4 Weed resistance to herbicides

While the use of herbicides in production agriculture was revolutionary and led to several-fold increases in crop yields, these compounds generated complications, in large part due their modes of action and chemical structures. One major problem with herbicide use and overuse is the development of weed resistance due to selection for mutants. This is the same phenomenon that occurs with use of antibiotics in humans (Dodds, 2017). To overcome this problem, manufactures produce herbicides that exploit several chemistries with differing modes of action. The concept is straightforward: the simultaneous evolution of various characteristics will occur only rarely in the same population. For example*, Conyza* is a cosmopolitan weed characterized by several herbicide resistances due to the pyramiding of point mutations. Only individuals that carry different mutations could survive combinations of herbicides with differing modes of action, including sulfonylurea or imidazolinone plus atrazine (Matzrafi et al., 2015).

Other controls over resistance of weeds include increases in the concentration of active ingredients and bundling of highly resistant plant lines with specific herbicide use. This has been the strategy for the use of Roundup (glyphosate) resistant crop plants with herbicide application. This idea may have also led to herbicide overuse.

## 5 Herbicides and Their Fate in the environment

The chemical structures of the active ingredients in herbicide formulations differentially interact with environmental matrices such as soil, sediments, particles, water, or with microorganisms that may degrade these compounds. These interactions may have major impacts on either the fate of the chemicals in the environment, their routes of degradation, or the formation and bioavailability of more toxic metabolites. For example, the herbicide 2,4-D (2,4 dichlorophenoxy acetic acid) is an organic acid with a systemic mode of action that has been used worldwide to control broad-leaf weeds in grass, wheat, rice, corn, sorghum, and sugarcane. This herbicide is translocated through the plant and accumulates in roots, stopping its growth. This herbicide, and its most commonly known degradation product, 2,4-dichlorophenol (2,4-DCP), are very soluble in water, and can be found in rivers and lakes, or in groundwater, even if it has not been used for long periods (Silva et al., 2007). This is one of the main reasons for discovering efficient degradation processes for 2,4-D.

In addition to resistance, herbicide use and overuse have led to the production of degradation product metabolites in the environment, including aminomethylphosphonic acid, a metabolite of glyphosate. This compound persists in soil, water, and plants with potential toxicological problems caused by the accumulation of residues in the food chain. The US Environmental Protection Agency (EPA) describes glyphosate as practically non-toxic and concluded that it was not an irritant under the acute toxicity classification system. Nevertheless, data regarding the toxic level of this herbicide have been generated on the basis of its mode of action in the shikimic acid pathway that is used for the production of amino acids in a small number of organisms, most of which are green plants (Bai and Ogbourne, 2016).

Samples analyzed in Hungary from 1990 to 2015 showed systematic contamination in watercourses by herbicides such as trifluralin, atrazine, diazinon, acetochlor and (more recently) glyphosate (Székács et al., 2015). Glyphosate has also been detected as a contaminant in groundwater, drinking water, and in urine of farmers in Mexico (Rendón-von Osten and Dzul-Caamal, 2017). The herbicides atrazine, ametryne, aetolaehlor, simazine, acetochlor, metolachlor and alachlor were detected in tap, surface, and groundwater samples in China (Li et al., 2018).

## 6 Herbicides affecting non-target organisms

**Figure 1:**
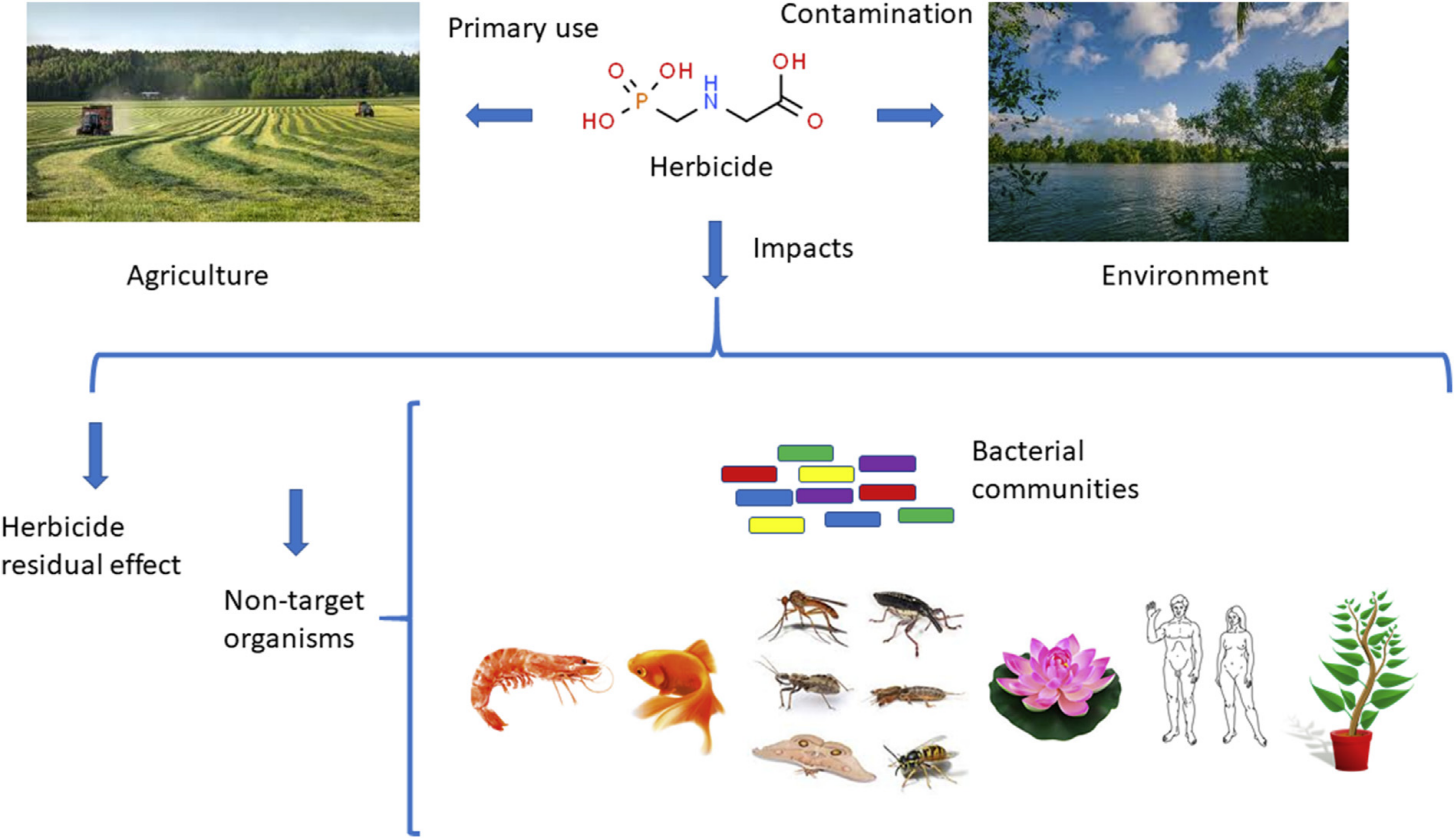
Impacts of the overuse of herbicides on agriculture and the environment affecting non-target organisms.

A search of the National Center for Biotechnology Information PubMed website, on September 9, 2019, using the keywords "herbicidal effects" and the major kingdoms of living things, recovered 14,511 papers for bacteria, 77 for archaea, 5,757 for protozoa, 21,408 for plantae, 13,875 for fungi, and 63,408 for animals. Importantly, a search of “herbicide and humans” recovered 41,245 papers. The structure and mode of action of active ingredients present in herbicide formulations are not specific to killing weeds, due in part to the high number of electronegative residues in their molecules, including oxygen, hydroxide, sulfonyl, phosphoric acid, amine, and chlorine. As such, these herbicides have high oxidative potential across various chemical targets and organisms, in addition to microorganisms (Figure 1). For example, atrazine has been postulated to exert some indirect effects over non-target organisms such as shrimp by inducing an oxidative stress response through enhanced peroxide production, as well as the induction of superoxide dismutase (SOD), glutathione-S-transferases, and glutathione reductase. These organisms possess systems to overcome these problems by activating antioxidant responses; however, there is an energy cost because there was a decrease in lipid storage in these animals (Griboff et al., 2014). Despite this evidence, the proposed impact of atrazine on non-plant biota remains controversial and without substantial scientific support.

Various non-target organisms can also be directly influenced by the electronegative properties of some herbicides. The long-term use of thiobencarb caused imbalances in agricultural soils and in aquatic systems, mainly due to its toxicity in invertebrates, fish, and microorganisms, reducing their number and diversity (Chu et al., 2017). A similar change in aquatic and agricultural ecosystems was seen after long-term application of chloroacetamide herbicides (Dong et al., 2015). The world-wide use of the herbicide 2,4-D has also impacted groundwater on account of its high solubility.

One simple explanation for why non-target organisms are adversely affected by herbicides is due to their ubiquity in the environment, such that many organisms cannot escape exposure. This is the case for the herbicide butachlor that was reported to negatively impact zebrafish in a dose-dependent manner, a commonly used aquatic model for early-life stage toxicity evaluation of environmental contaminants. Butachlor also caused enhanced production of ROS and malondialdehyde in zebrafish (Xiang et al., 2018).

Herbicides in antifouling paints also appear to produce alterations in non-target species. These paints are used to prevent the attachment of organisms to submerged surfaces of vessels and aquatic structures. The active ingredients, which in some cases have the composition of broad-spectrum herbicides and fungicides, are released from the coated surface and protect the surfaces. One of the antifouling components, tributyltin, is so persistent, that even though its use was banned in 2008, it continues to be found in the environment. Alternative biocides for anti-fouling paints, such as irgarol (2-methylthiol-4-tert-butylamino-6-cyclopropylamino-s-triazine) and diuron also have widespread distribution in oceans, and may cause harmful effects despite their low concentrations (Manzo et al., 2014). Compound M1 (2-methylthio-4-tert-butylamino-6-amino-s-triazine) a by-product of metabolism of irgarol, and the parent compound are bioaccumulated by aquatic plant species in marine environments (Fernandez and Gardinali, 2016), contributing to the persistence of these contaminants.

Similarly, the long-term use of the herbicides alachlor, acetochlor, butachlor, and metolachlor also caused imbalances in aquatic and agricultural environments. This is important for public health because the US EPA has suggested that acetochlor may have carcinogenic potential (Wang et al., 2015).

In some instances, herbicide over-application has negatively impacted soil microbiota, affecting the dynamics of biogeochemical cycles and soil fertility (Elias and Bernot, 2014), likely due to loss of sensitive microbial populations providing specific ecological functions. Nevertheless, the chemical structures of herbicides may provide essential nutritional components for the growth of microorganisms. An additional effect of overuse of herbicides is the impact that they have on soil microbial community structure and composition, with secondary influences on plant nutrition and herbicide sensitivity. This is in large part due to impacts on the functions of microorganisms in mutualistic interactions with plants. Herbicides can affect the metabolism of plants, altering exudates used for signaling in plant-microbe interactions. This was shown in the model plant *Arabidopsis thaliana* exposed to the herbicide imazethapyr. Application of the herbicide resulted in changes in root cell wall structure and increased citrate production and exudation. These changes were thought to subsequently modify microbial community structure in the rhizosphere microorganisms, and to alter root morphology (Qian et al., 2015).

This is a fundamental reason to focus attention on the excessive use of herbicides in agriculture; they sometimes exert toxic effects that travel up the entire food chain. “As pointed out by Rachel Carson in her book “Silent Spring”, pesticides are not only toxic to their intended primary targets, but also to non-targets, resulting in ecological imbalances (Carson, 2002).” (Carson, 2002).

## 7 Bacterial herbicide resistance systems that do not involve biodegradation

Microorganisms that are important for maintaining soil fertility can be affected by oxidative stress caused by the electronegativity of the chemical structures making up the active ingredients of herbicides. While the primary purposes of herbicides are to damage or kill target weeds, they can provoke oxidative stress in a variety of non-target organisms through the production of free radicals. While inhibiting metabolic pathways in weeds, the active molecules of the herbicides generate ROS, thereby affecting enzymes in non-target organisms as well. More specifically, the primary effect of herbicides that alter photosynthetic systems can affect plants other than their primary targets, as well as affecting photosynthetic cyanobacteria. In some situations, metabolic intermediates of herbicide degradation may be toxic to non-target organisms, possibly maintaining electronegative residues in their molecular structures. Such is the case for degradation residues of quinclorac, which are phytotoxic to many crops, vegetables and microorganisms (Liu et al., 2014).

Studies done using antioxidative enzymes have demonstrated their effectiveness in allowing some microorganisms to overcome toxicity due to herbicides. For example, Gravina et al. (2017) evaluated the influence of paraquat on the physiology and adaptive capacity of mutant strains of non-target *Escherichia coli*, knocked out in the Mn-SOD (*sodA*) and Fe-SOD (*sodB*) genes. SOD is an ancient enzyme that evolved to adapt to oxidative atmospheres. The metals Fe and Mn are important for enzyme stability and catalysis, as well as for the overall structure of the enzymes. These enzymes possess significant differences in their oxidation and reduction potentials and may have provided significant advantages to organisms in variable O_2_ and heavy metal environments (Case, 2017). Therefore, mutations in the SOD genes may alter metabolism and antioxidative responses of these strains, through generation of new isoforms that vary according to the oxidative conditions generated by the herbicides. This versatility is a good model of phenotypic plasticity, leading to adaptation to herbicide. This model can be found in several organisms, including the marine ciliate *Euplotes focardii* that lives in Antarctica within a very narrow temperature range (4–5 °C) (Pischedda et al., 2018). According to these authors, a major issue for this organism is the oxidative stress due the substantial amounts of dissolved oxygen that characterize Antarctic marine environments. The origin of these isoforms can be derived from gene duplication and diversification. This diversification probably occurred by independent mutations and selection pressure.

Another example of an antioxidative system responding to herbicidal toxicity is found in *Pantoea ananatis*, isolated from agricultural soil. This bacterium resists and grows in the presence of mesotrione, likely due to the presence of a polymorphic catalase (CAT) enzymes controlling oxidative stress. Bacteria resistant to mesotrione show changes in lipid membrane saturation, likely leading to increased membrane impermeability, and enhanced formation of glutathione-s-transferase-mesotrione (GST-mesotrione) conjugates, enhancing herbicide degradation levels (Prione et al., 2016). Structural changes are also related to herbicide-induced stress tolerance. Changes in the membrane lipid saturation pattern in bacteria can act as selective barriers against herbicides (Dobrzanski et al., 2018; Prione et al., 2016; Rodríguez-Castro et al., 2019). In this review, we use the term “resistance” to refer to the ability of bacteria to grow in the presence of herbicides, irrespective of duration of treatment (Brauner et al., 2016).

Various enzymatic and non-enzymatic systems act in response to the oxidative effects of herbicides, as is the case for the cyanobacterium *Synechocystis,* which responds to arsenite and arsenate via induction of general stress responses, induction of redox scavenging systems and chaperones, and by repression of genes involved with photosynthesis and growth (Sánchez-Riego et al., 2014). In addition, arsenic is present in herbicides such as monosodium methylarsenate, as trivalent arsenicals that react with thiol groups in proteins and inhibit various biochemical pathways; there is no specific target for this herbicide. Trivalent arsenicals interfere with small molecule thiols such as reduced glutathione, resulting in the production of ROS and oxidative stress (Chen et al., 2015).

The hazardous effects of herbicides on non-targeted microbes and plants can be mitigated through the accumulation of stress metabolites such as poly-sugars, proline, glycine-betaine, and abscisic acid, and through upregulation in the synthesis of enzymatic and non-enzymatic antioxidants such as SOD, CAT, ascorbate peroxidase (APX), glutathione reductase, ascorbic acid, α-tocopherol, and glutathione (Gouda et al., 2018). High levels of thioredoxin, glutaredoxin and GPX were associated with atrazine stress in the interaction between the mycorrhizal fungus *Glomus mosseae* and alfalfa (*Medicago sativa*) (Nath et al., 2016).

## 8 Bacterial nonspecific responses to herbicides

Several responses systems in bacteria are not herbicide-specific but are rather related to other stressful substances. These nonspecific response systems enhance the survival and fitness potential of these organisms. For example, bacteria respond to certain environmental stresses by altering the transcription of regulons, thereby enabling the cell to cope with the stress. The same operons however, may also be regulated by different stresses, as in the case of antibiotics and herbicides; as is the case of paraquat inducing resistance to norfloxacin in *E. coli* (Rosner and Slonczewski, 1994). The same is true for the herbicides dicamba, 2,4-D, and glyphosate, that at sub-lethal doses were found to induce changes in *soxS-lacZ* fusion strains of *E. coli* and *Salmonella enterica* in response to antibiotics. This regulon is responsible for the upregulation of efflux pumps and reduction of porins, enhancing antibiotic resistance (Kurenbach et al., 2015). Herbicides and other chemicals used in agriculture and domestic gardens can induce phenotypes akin to multiple-antibiotic resistance in potential pathogens faster than the lethal effect of the antibiotics. The combined use of both herbicides and antibiotics near farm animals and insects like honeybees might lead to an immediate decrease in their therapeutic usefulness, eventually leading to even greater use of antibiotics (Kurenbach et al., 2015).

While some bacterial responses to herbicides are specific, such as induction and modulation of antioxidant enzymes and herbicide degradation genes, others generate nonspecific responses that lessen secondary damage to cellular functions. For example, the herbicide Callisto was shown to induce changes in lipid saturation and membrane permeability in *Bacillus megaterium* strains isolated from various agricultural environments (Dobrzanski et al., 2018). Complementary routes to obtain energy can also be used to reduce the toxicity of herbicides. *P. ananatis,* for example, can degrade mesotrione, the active ingredient of the herbicide Callisto, but without using it as a carbon, nitrogen, or sulfur source for growth. For this bacterium, mesotrione catabolism required glucose supplementation (Pileggi et al., 2012).

Herbicide degradation may also be hampered by collateral effects due to exposure of bacteria to toxic metals in environment, leading to accumulation of intracellular ROS, and the consequent upregulation of genes related to herbicide degradation. For example, the soil bacterium *Cupriavidus pinatubonensis,* when exposed to sub-lethal concentrations of copper, increased the concentration of ROS. As a result, there was upregulation of an Ohr/OsmC family member protein, subsequently affecting the degradation of phenoxy acid herbicides (Svenningsen et al., 2017). There are also reports of non-enzymatic systems in the control of ROS. Such is the case for the role of up-regulated genes encoding for spermidine production, which contribute to the survival of *Burkholderia pseudomallei* in stressful environments, mainly under physiological and oxidative stress conditions (e.g., hydrogen peroxide) (Jitprasutwit et al., 2014).

Perhaps a better example of nonspecific responses can be seen in the case of superoxide stress that leads to the production of ROS. In order to survive under these conditions, cells must coordinate regulation of a variety of metabolic pathways. One major adjustment is via increased production of NADPH and a concomitant decrease in NADH generation in *E. coli* (Rui et al., 2010). In this case cellular strategies which maximize survival under stress conditions takes precedence over metabolic efficiency.

## 9 Bacterial herbicides degradation pathways and bioremediation

Many microorganisms utilize herbicides as sole sources of nutrients for growth and survival in the environment. The process of natural selection has undoubtedly improved fitness of microorganisms harboring herbicide degradation genes. This has led to some positive aspects of herbicide effects on microbial diversity. This review focuses on the study of the great biochemical diversity associated with phylogenetic diversity (Weissenbach, 2019) that can therefore be the basis for the wide system of responses to herbicides in bacteria. The application of the herbicides from the thiocarbamate, dinitroaniline, and chloroacetamide families increased microbial biomass, measured by the chloroform fumigation method, probably due to direct degradation or via co-metabolic processes. This increased the availability of mineral carbon, nitrogen, and phosphorous to the soil and resulted in higher mineralization of these herbicides (Barman and Das, 2015). Chloroacetamide herbicides can be transformed by microbial metabolism in natural soils to 4,2-methyl-6-ethylaniline, and this intermediate can be used as a sole nutrient source for a *Sphingobium* strain. This intermediate can also undergo a series of enzymatic reactions, resulting in the production of 2-methyl-6-ethylhydroquinone and 4-hydroxy-2-methyl-6-ethylaniline. The horizontal transfer of genes encoding enzymes involved in these degradative pathways in bacteria is probably important for the survival of these organisms in polluted environments (Dong et al., 2015).

## 10 Major herbicides degradation pathways

**Figure 2:**
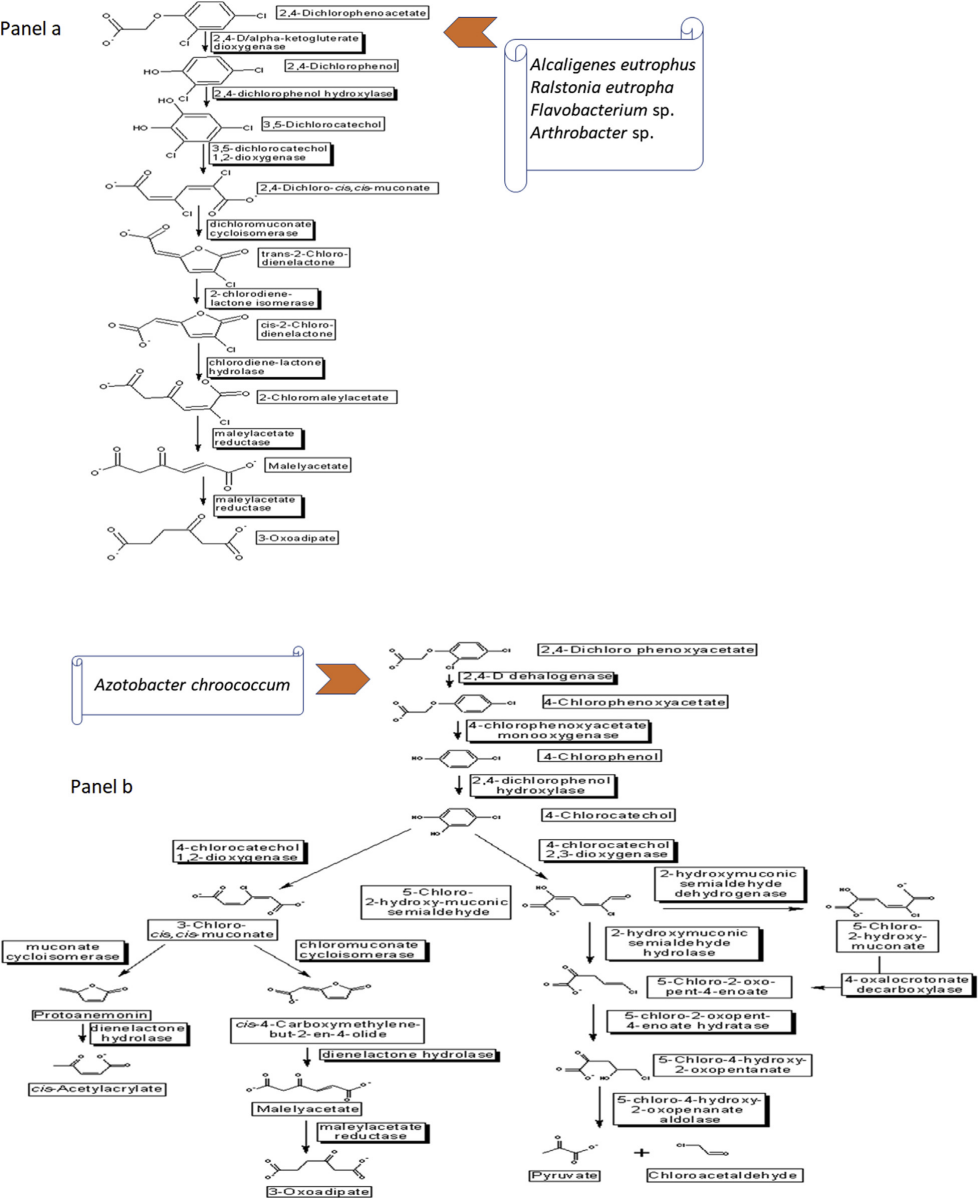
2,4-D degradation routes, according to Biocatalysis/Biodegradation Database (Gao et al., 2010). The microorganisms indicated in the figure are some of those responsible for initiating the metabolic degradation routes (panels a and b), but other species may be responsible for the other steps.

Strategies to reduce 2,4-D contamination in agricultural soils have been tested using bio-augmentation techniques that did not show good efficiency on account of the low survival rate of degrading strains, because laboratory conditions cannot reproduce the stressful conditions of the natural environment. An alternative would be the introduction of plasmids containing 2,4-D degradation genes into indigenous bacteria, which are well adapted to the environment where bioremediation will be performed (Kumar et al., 2016). The degradation of 2,4-D occurs by two well-known metabolic pathways, with several enzymes and microorganisms already described with this ability (Figure 2).

The impacts of herbicides on microbial consortia may also reflect evolutionarily-selected organizations to optimize specialization and sharing of metabolic routes. A microbial consortium, mainly containing the genera *Bacillus*, *Phyllobacterium*, *Pseudomonas*, *Rhodococcus*, and *Variovorax,* could use azimsulfuron as the sole nutrient source, degrading the herbicide better together than what was achieved using isolated pure cultures. This is likely due to complementary (synergistic) metabolism among bacterial consortia members for the degradation of the herbicide (Valle et al., 2006).

**Figure 3:**
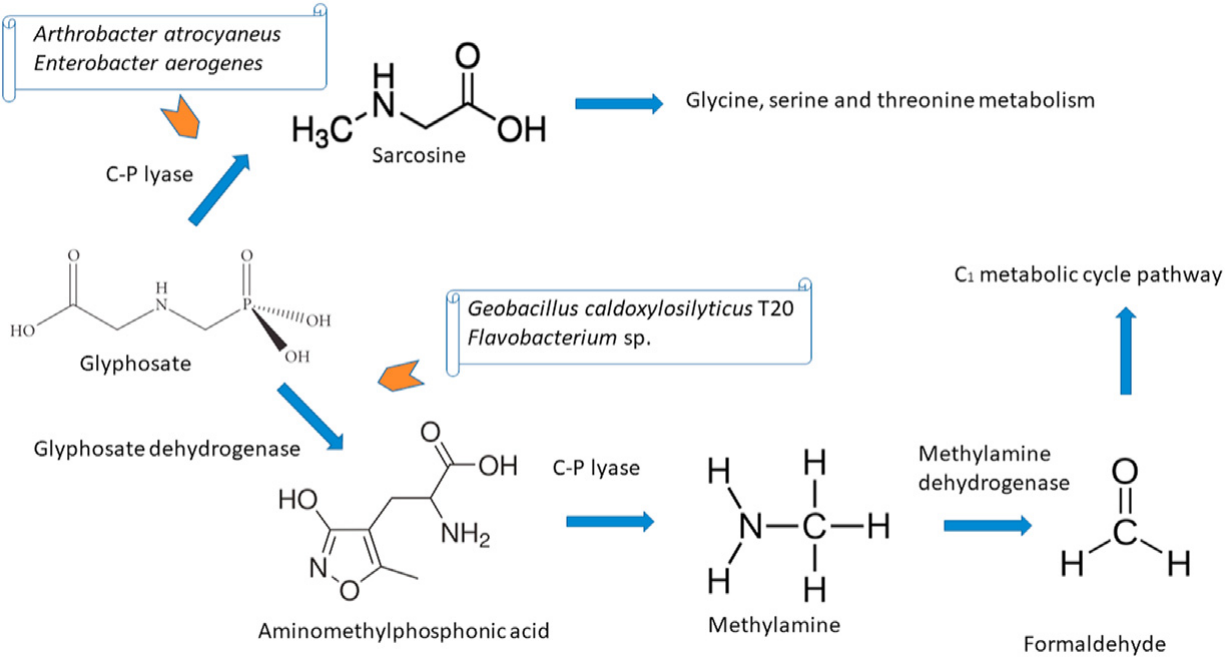
Glyphosate degradation routes, according to Sviridov et al. (2015) and Biocatalysis/Biodegradation Database (Gao et al., 2010). The microorganisms indicated in the figure are some of those responsible for initiating the metabolic degradation routes; however, other species may be responsible for the other steps.

Glyphosate is degraded by 19 bacterial and five fungal species, via at least two distinct metabolic routes. In the route where a sarcosine intermediate was found, degradation genes were organized into the *phn* operon, encoding a C–P lyase (Sviridov et al., 2015) (Figure 3). In the systems where the aminomethylphosphonic acid (AMPA) intermediate was found, the *glpA* (homologous with hygromycin phosphotransferase genes) and *glpB* genes are involved. Other genes related to this degradation route involve the glyphosate oxidoreductase (*gox*) gene, responsible for the transformation of this herbicide into glyoxylate and its major degradation product AMPA. Herbicide-resistant transgenic crops were obtained by transformation with these genes (Huang et al., 2017). Due to the high toxicity of glyphosate and AMPA, the bioremediation process needs to be performed on biosafety compounds. Routes based on C–P lyases have low efficiency because this enzyme is inactivated under field conditions (Figure 3). Another difficulty is the search for combinations of strains that mineralize this herbicide faster, especially to prevent the accumulation of toxic intermediates (Sviridov et al., 2015). Despite the idea of transforming indigenous bacteria with these degradation genes, thereby obtaining bioremediating microbes already adapted to the contaminated sites, we believe that more sustainable processes are based on the assembly of bacterial consortia.

**Figure 4:**
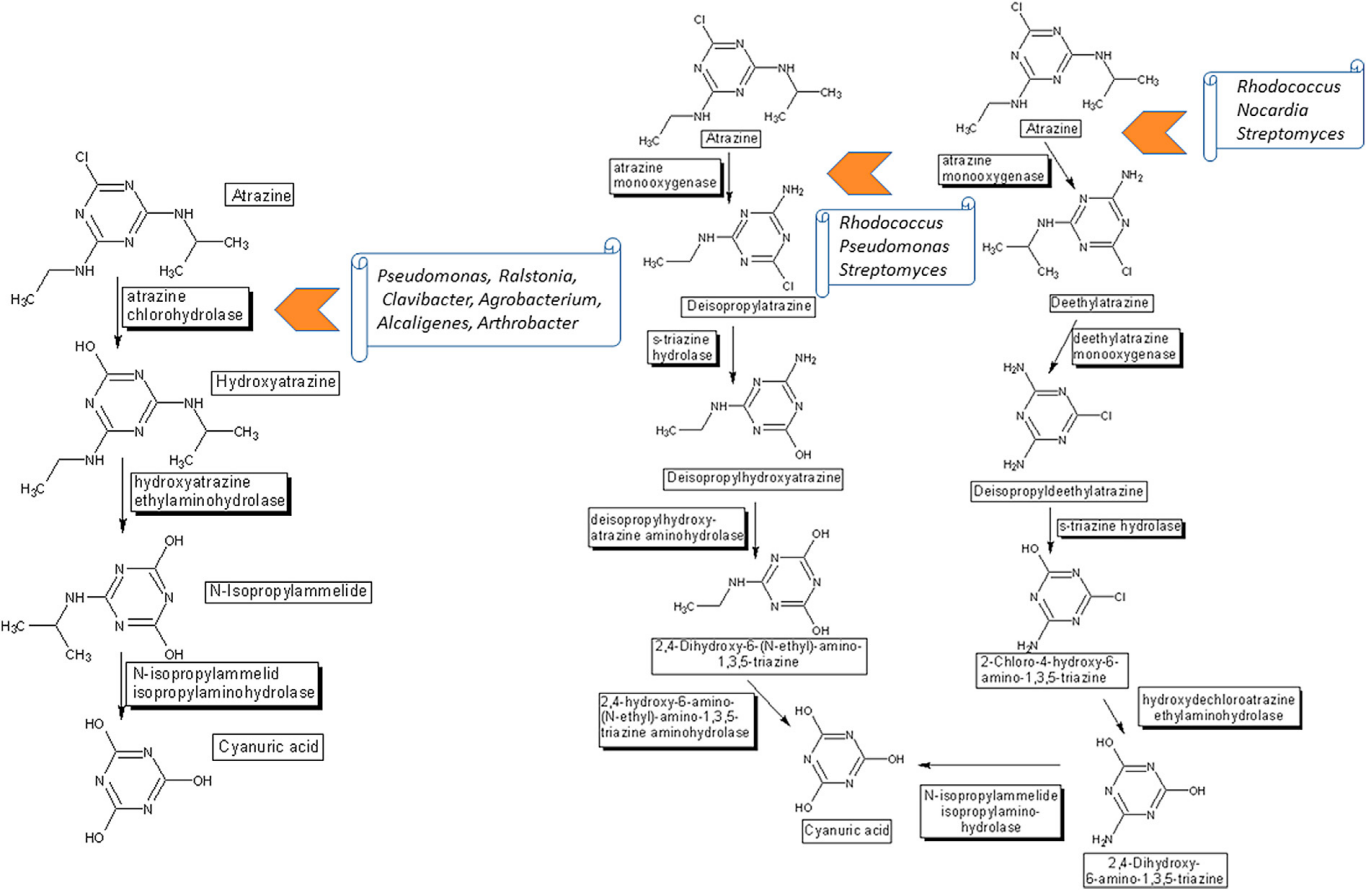
Atrazine degradation routes, according to Sadowsky et al. (1998) and Biocatalysis/Biodegradation Database (Gao et al., 2010). The microorganisms indicated in the figure are some of those responsible for initiating the metabolic degradation routes; however, other species may be responsible for the other steps.

Bacteria such as *Pseudomonas* ADP and *Arthrobacter aurescens* have acquired the ability to metabolize atrazine, but only after six or so genes were acquired by each species (Martinez et al., 2001; Mongodin et al., 2006). In some rare cases, the evolutionary pressure may result in the assembly of all pathways for herbicide degradation in a single bacterium, as was the case for *Pseudomonas* ADP. This strain harbors all genes required for the compete degradation of atrazine (Sadowsky et al., 1998) (Figure 4). There are now numerous reports of specific routes of herbicide degradation, leading to the belief that these systems were selected for after contact with the agent. Nevertheless, even these routes are related to the degradation of structurally similar herbicide families, because *de novo* gene conversion is a rare event. For example, AtzB is a key enzyme in the metabolic pathway for s-triazine biodegradation. AtzB is essential for microbial growth on s-triazine herbicides and is responsible for the hydrolytic conversion of hydroxyatrazine to N-isopropylammelide (Martinez et al., 2001). The AtzB enzyme contained conserved mononuclear amidohydrolase superfamily active-site residues. Substrates for this enzyme require a monohydroxylated s-triazine ring, with at least one primary or secondary amine substituent, and either a chloride or an amine leaving group. Consequently, the enzyme catalyzes both deamination and dichlorination reactions (Seffernick et al., 2007). Due to its composition with several nitrogen atoms, nitrogen fertilization may affect the degradation rates of this herbicide in agricultural soil. The addition of carbon sources may induce the increase of populations harboring plasmids containing atrazine degradation genes, placing bioaugmentation as an alternative for mitigating contaminated soils (Singh and Singh, 2016).

Microbial consortia in biofilms function to mineralize organic xenobiotic compounds, possibly by the sharing of metabolic routes by different species and optimization of the production and consumption of energy in metabolically-integrated communities. Such is the case for metabolic association among the proteobacteria *Variovorax* spp., *Comamonas testosteroni*, and *Hyphomicrobium sulfonivorans*. Together, this consortium converts the phenylurea herbicide linuron into products that are degraded by other bacteria in the consortium. In its presence, the gene encoding linuron hydrolase, *hylA*, and others contributing to carbohydrate, amino acid, nitrogen, and sulfur pathways showed significantly increased expression. It appears that the *Variovorax* strain indirectly gained nutrients and energy from linuron by metabolizing excretion products produced from the *C. testosteroni* and/or *H. sulfonivorans* strains. The *Variovorax* strain also had an elevated stress response and overexpressed genes involved in cell-to-cell interaction systems, such as quorum molecule signaling and type VI secretions. The latter two systems could be used by *Variovorax* in interference competition with *C. testosteroni* and *H. sulfonivorans* (Albers et al., 2018).

There are other examples where metabolically-integrated microbial communities have shown great potential for degrading a wide variety of herbicide substrates. A novel thiobencarb degradation pathway has been proposed for an *Acidovorax* strain. This bacterium oxidized and then cleaved the C–S bond of thiobencarb, producing diethylcarbamothioic S-acid and 4-chlorobenzaldehyde. These products were subsequently oxidized to 4-chlorobenzoic acid and then hydrolytically-dechlorinated to 4-hydroxybenzoic acid by other strains (Chu et al., 2017). Another example is herbicide biodegradation by *P. ananatis* that proceeds through to the formation of GST-mesotrione conjugates, enhancing herbicide degradation levels (Prione et al., 2016). While most bacteria degrade mesotrione via 2-amino-4-methylsulfonyl benzoic acid or 4-methylsulfonyl-2-nitrobenzoic acid, recent LC–MS/MS analyses indicated that biodegradation of mesotrione by other microorganisms leads to the formation of novel intermediates (Pileggi et al., 2012).

The transformation of herbicides in soils do not only involve free-living microbes, which are likely few in soil systems. The selective effect of herbicides may also favor the interaction between endophytic bacteria and their host plants grown on commercial farms. For example, the biodegradation of quinclorac in natural settings is relatively slow and transformation residues are toxic to many crops, vegetables, and microorganisms. An endophytic *B. megaterium* strain obtained from the roots of tobacco degraded 93% of quinclorac in 7 days. The degradation products were different from those presented in previous publications, suggesting that this bacterium uses novel routes for the degradation for quinclorac. Studies of tobacco grown in pots suggested that *B. megaterium* alleviates quinclorac phytotoxicity (Liu et al., 2014).

### 10.1 Bioremediation approaches

In addition to their effects on free-living soil microorganisms, the impacts of herbicides on the environment can also be mitigated using endophytic bacteria, those living within plant tissues that are capable of herbicide degradation. Endophytic strains may contribute to the survival of both agricultural and weed plants in herbicide-contaminated environments via xenobiotic degradation pathways (Tétard-Jones and Edwards, 2016). A similar concept was tried in the past for control of corn borer by using an endophytic *Clavibacter xyli* subsp. *cynodontis* adapted to plants containing the *cry* gene, which encodes a toxin with effects against insects (Fahey et al., 1991). In this manner, agricultural products would be protected against insect attacks through the metabolites produced by an endophytic strain. Liu et al. (2014) used this same strategy to transform quinclorac and to identify its metabolites; however, in this context, endophytic strains protect against the toxic effects of herbicides. This herbicide, used to control several grass species in rice, canola, barley, corn, and sorghum, is degraded by the endophytic *B. megaterium* strain Q3.

Owing to the plasticity of metabolic pathways in bacteria, their use for bioremediation is one key method of addressing these issues, even using a classically non-environmental bacterium such as *Escherichia coli*. The *E. coli* strain DH5-α was found to degrade the compound mesotrione (2-(4-methylsulfonyl-2-nitrobenzoyl) cyclohexane-1,3-dione) in only 3 h without previous exposure to the herbicide (Olchanheski et al., 2014). Mesotrione is the active ingredient of the herbicide Callisto, used for control of weeds that grow in maize crops. This active ingredient is synthesized from a phytotoxin found in the plant *Callistemon citrinus* that inhibits the enzyme 4-hydroxyphenylpyruvate dioxygenase, which converts tyrosine to α-tocopherol and plastoquinone. Inhibition of the latter leads to a decrease in synthesis of carotenoids, resulting in tissue death (Olchanheski et al., 2014).

There are several technologies aimed at eliminating herbicides in the environment, mainly from water. There are systems based on adsorption onto iron composite nanoparticles (Ali et al., 2016), absorption by graphene nanosheets (Kamaraj et al., 2017) and bioremediation. Despite the advanced technologies, herbicide contamination in drinking water remains a worldwide problem (see “Herbicides and Their Fate in the Environment”). There are options for treatment; however, current strategies have proven to be ineffective in remediating water.

**Figure 5:**
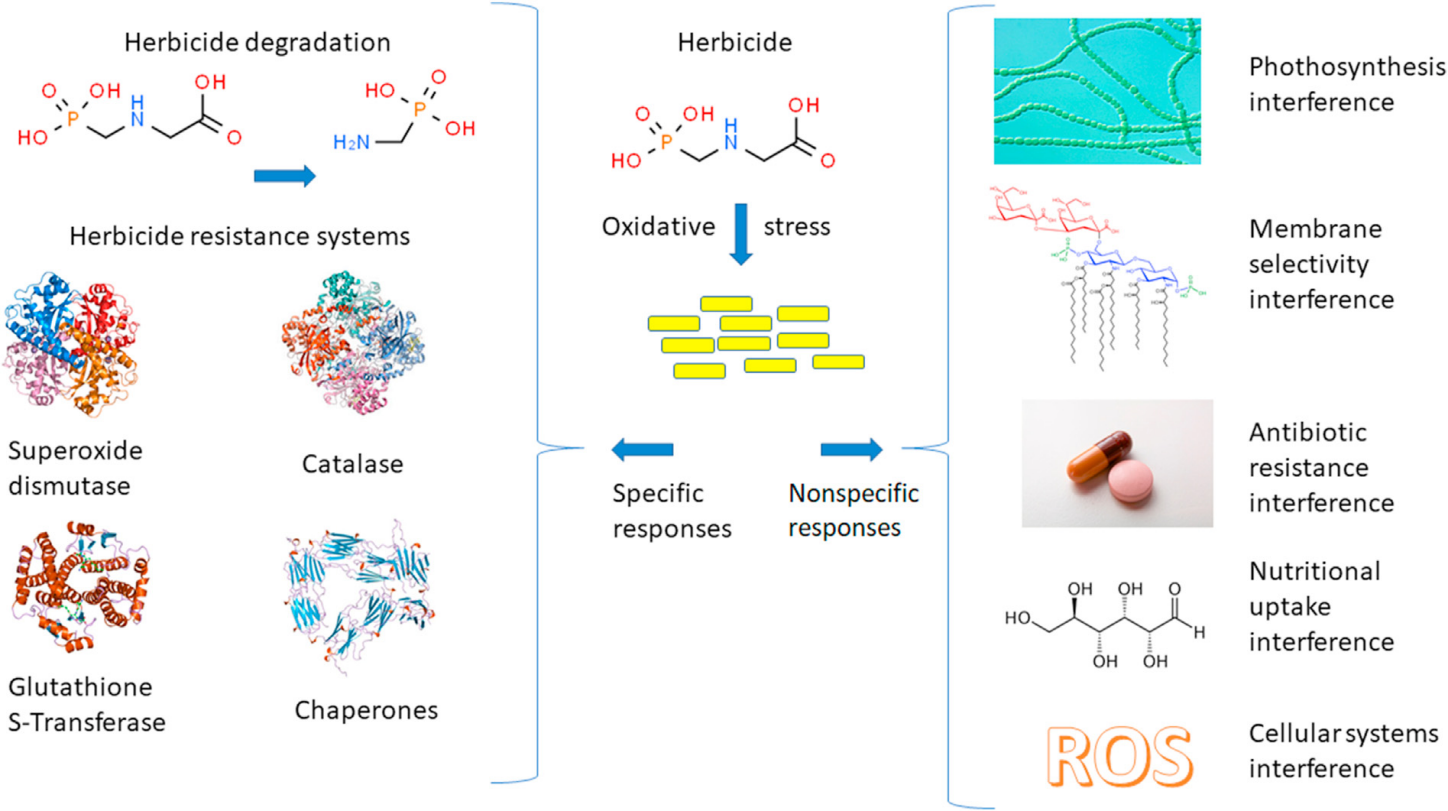
Influence of herbicides on specific and nonspecific responses systems of bacteria.

Bioremediation is a complex process because it is related, as described in this article, to resistance to toxic substances through general systems involving structural and enzymatic systems (Figure 5). The degradation metabolic pathways involve various steps and different routes, with the participation of various species of microorganisms possessing interconnected degradation networks (Figures 2, 3, and 4) that are organized in biofilm consortia via chemical quorum sensing signaling. This type of study is overly complex and requires molecular approaches, which will be discussed in the next section.

## 11 Bacterial xenobiotic responses by omics-based approaches and perspectives for bioremediation technologies

Modern high-throughput techniques of molecular analysis, the omics-based approaches, generate very large amounts of data regarding taxonomies and genetic structures of bacterial communities, potential functional capabilities, and stressor responses that can be explored more efficiently with the help of bioinformatic tools. These approaches include methods such as gene amplicon sequence (sequencing of a gene or gene fragment of an entire community), shotgun metagenomics (sequencing of community DNA), metatranscriptomics (analysis of mRNA profile in a community), proteomics (proteins present in a biological sample), and metabolomics (metabolites present in biological samples (Rebollar et al., 2016). According to these definitions, metaproteomics can be understood as a set of techniques that allows the study of a community's set of proteins in certain environments, allowing associations between gene expression and adaptation (Gutleben et al., 2018). Therefore, omics-approaches can be understood as methodologies designed to understand the dynamics of molecules related to gene expression and metabolism of an entire cell or community. In this context, the plant *Arabidopsis thaliana* was exposed to trace concentrations of the S- and R-imazethapyr enantiomer to examine herbicide toxicity effects on root proteome via iTRAQ-based quantitative studies. Computational, physiological, and metabolic analyses showed that imazethapyr reduced branched chain amino acid content in tissues by strongly suppressing their synthesis and by increasing their catabolism (Qian et al., 2015).

### 11.1 Sequencing approaches

Traditional techniques in environmental microbiology have facilitated the study of metabolic and genetic associations in communities of microorganisms structured in biofilms. Nevertheless, new molecular techniques and bioinformatics approaches achieve these same goals much more effectively. Next-generation Illumina transcript-sequencing technology (RNAseq) that allows analysis of global gene expression between strains, has been used to identify potential genes related to interspecies interactions. This technique has proven to be especially useful in examining microbial consortia present in biofilms that have the ability to transform (mineralize) xenobiotic compounds such as the phenylurea herbicide linuron (Albers et al., 2018). Nevertheless, it is important to keep in mind that there are novel methods beyond omics-based techniques that have been used to study bioremediation and biodegradation. For example, the microbial electrochemistry based in transfer of electrons between cells and electron conductors such as naturally occurring minerals or solid-state electrodes can be used to remove oxidized and reduced pollutants from the environment in a bioremediation process called microbial fuel cell (Wang et al., 2020).

Historically, the application of microorganisms for bioremediation processes occurs after advancing studies of degradative metabolism. For example, Vilela et al. (2018) describe a variety of microorganisms that can degrade endocrine-disruptor compounds, a class of hormones considered to be hazardous pollutants; however, degradation must occur completely so that even more hazardous metabolites are not produced. However, more efficient levels of technical development in the elimination of xenobiotics can be obtained using omics-based technologies, since it provides information on the interrelationships between metabolic routes in bioremediation communities. This ultimately can provide needed information on possible ecological impacts. Such was the case when RNAseq-based differential transcriptomics were used to examine consortia gene expression occurring during linuron degradation (Albers et al., 2018). This approach led to discovery of previously uncharacterized proteins with functions relevant to cellular performance.

### 11.2 Genomes approaches

Several issues remain controversial in environmental microbiology, including the identification of bacterial species and the issue of whether the decrease in bacterial diversity is related to loss of soil functions. There are methodological questions about the reliability of microbial diversity and functionality assessments (Nannipieri et al., 2017). These are issues that we are considering in this review, mainly related to the effectiveness of bioremediation programs and their ecological sustainability. These are challenges that omics-based technologies have faced. In this sense, Thavamani et al. (2017) anticipated that ecological recovery in post-mining processes would occur after the introduction of plant-specific microbial consortia for synchronized plant-microbial remediation. In that case, the identification of contaminants and biodegrading microorganisms was essential.

Functional metagenomics combined with gene expression experiments allows the description of genes displaying unknown functions in isolation. This approach also can be used for previously assigned functions that had never been shown to be involved with the subject under study. This was the case for arsenic, for which the microbial communities from the Tinto River, a natural acid mine drainage site, were explored to search for novel genes involved in arsenic resistance (Morgante et al., 2015). The predicted metagenomics bioinformatics analysis was used in biofilm and planktonic communities in reservoirs containing herbicide-contaminated wastewater to characterize important genes, which functions were relevant for survival in these environments, by performing only 16S rDNA amplicon next-generation sequencing, and analyzing the genes present in the identified OTUs. With this technique it was possible to identify genes functions related to biofilm formation and structure, membrane transport, quorum sensing and xenobiotic degradation (Lima et al., 2020).

Omics-based approaches are also interesting for the rare biosphere. This consists of bacterial, archaeal, and fungal species that occupy an exceedingly small segment of the microbial communities in soil and water environments. While low in numbers, these rare microbes may be functionally important and are inherently difficult to study even through molecular approaches. For example, Wang et al. (2017), studied water samples from Lake Lanier, located at the northern part of the state of Georgia, USA, used as a drinking water reservoir. These authors added 40 μM of 2,4-D, among other chemicals, to samples and considered this to be a perturbation of the chemical quality of water. The population of degraders of organic compounds such as 2,4-D that are rarely detected in these environments by quantitative PCR techniques (qPCR) or metagenomic sequencing increased significantly in abundance following the environmental perturbation. Data obtained from sequence analyses of various isolates with degradation capacity, or from metagenomes, showed that differing co-occurring alleles of degradation genes are often transmitted on plasmids. Studies also showed that several species dominated post-enrichment microbial communities. This genetic reservoir, represented by members of the rare biosphere, can often be missed in metagenomic analyses; nevertheless, they are important because they enable microorganisms to respond to organic pollutants.

Pyrosequencing of 16S rRNA gene amplicon and predicted metagenomic analysis were performed to identify species of microorganisms with higher potential for degradation of primitive electronic waste from aquatic contaminated environments. In this manner, new omics approaches could be used to detail potential genes related to the degradation of toxic organic pollutants and heavy metals associated with specific taxonomic units (Liu et al., 2018). Metagenomics also can help in the prospecting for herbicide degradation genes. With this intention, Jin et al. (2007) constructed a metagenomic library comprised of DNAs collected from soils from a glyphosate storage area with a 15-year herbicide contamination history. The library was screened by using an *E. coli* mutant harboring a kanamycin cassette within the *aroA* sequence encoding an enzyme in the shikimic acid pathway, 5-enoylpyruvylshikimate-3-phosphate synthase. As a result, this bacterial strain, sensitive to glyphosate, was unable to grow in a minimal medium with the herbicide, at least if a DNA fragment from the metagenomic library containing a gene encoding a glyphosate-insensitive enzyme was inserted in the mutant strain genome. Using this approach, a gene was fished out of the library that generated the ability to restore growth to the *aroA* mutant (Jin et al., 2007).

### 11.3 Metabolic approaches

Many bioremediation strategies are based on metabolic processes of isolated bacteria and sometimes fungi. However, some factors may hinder the application of these microbiota to many environments. One issue is that the metabolic processes may depend on communication among microbial communities organized in biofilms and may depend on quorum sensing. *C. testosteroni*, *H. sulfonivorans* and *Variovorax* spp. cooperate in biofilm structures in soil for the synergistic degradation of the herbicide linuron. None of these species alone was able to degrade linuron (Flemming et al., 2016). Thus, the speed of this process may differ between isolated strains and those in communities. Another factor is that the process may be incomplete, and the metabolites may be more toxic than the active molecules of the herbicides. Rather than studying this piecemeal, tolerance to oxidative stress, herbicide degradation, and other complex response systems may be better understood using omics-based approaches.

Other alternative strategy to improve xenobiotics bioremediation is through coordinating expression of genes encoding for degrading enzymes by quorum sensing systems. Quorum sensing is characterized by signaling molecules dependent on population density that control the behavior of various species of microorganisms that influence biofilm formation and metabolic pathways in coordinated fashion. According to this view, one strategy chosen to improve polycyclic aromatic hydrocarbon (phenanthrene and pyrene) bioremediation by *Pseudomonas aeruginosa* is via the coordinated expression of genes coding for degrading enzymes impacted by quorum sensing. These data were confirmed by using intercellular signaling acylated homoserine lactone bioreporters and GC-MS analysis (Kumari et al., 2016). A synthetic consortium of *E. coli* strains was designed to directly produce isopropanol from cellobiose, by metabolic paths sequentially coordinated by a synthetic quorum sensing system (Honjo et al., 2019). The ability to coordinate gene expression in different microbial species in cooperative response to environmental stimuli increases the ability to adapt to toxicologically-impacted environments.

Various molecular approaches have also shown the importance of genes related to communication, including quorum sensing and community structuring in biofilms. For example, genes encoding enzymes related to polycyclic aromatic hydrocarbon degradation were found in *P. aeruginosa* using a network analysis approach. Co-expression data from a publicly available database, the Gene Expression Omnibus, were used to uncover degradation genes under various stress conditions. As expected, no gene acted alone, and several stresses usually induced distinct metabolic pathways for degradation, quorum sensing, biofilm formation, and tolerance to antibiotics (Yan and Wu, 2017).

Another way of controlling the characteristics of microbial communities in structured biofilms is by the introduction of plasmids that control cell numbers. This strategy can be used with aerated and non-aerated membrane systems used in various water treatment operations, as well as in the food and power generation industries. Biofouling typically reduces flow and increases energy consumption in membrane-based systems due to the build-up of microorganisms in the polymeric matrices of biofilms. It may be possible to engineer the materials and bacteria to prevent biofouling by limiting bacterial cell numbers and consequently biofilm thickness. This concept is best exemplified by the engineering of a “beneficial” biofilm to encode an epoxide hydrolase. This enzyme can be used to degrade the xenobiotic epichlorohydrin, as well as limiting its own thickness by modulating a quorum sensing system and by secretion of nitric oxide. Epichlorohydrin is commonly used as a precursor for the synthesis of glycerin, epoxy resins, elastomers, pesticides, textiles, membranes, paper, and pharmaceuticals (Wood et al., 2016). To avoid issues of horizontal transfer of the genes involved in quorum sensing, coding sequences were integrated into the bacterial chromosome.

New methods using DNA, RNA, proteins, metabolites, metagenomes, and epigenomes have been used to elucidate the behavior of populations of various species under the influence of environmental contaminants. This is a shift from the standard biotechnological view of individual strains to one using biotechnology based on microbial communities, consortia, or biofilms. In studies on aquatic environments contaminated with hexavalent chromium, the resistant bacteria *Pannonibacter phragmitetus* BB was evaluated using a number of molecular approaches to define its multiple-response system, including enzyme activity assays, chemotaxis assays, genome sequencing, comparative genome analysis, proteomic analysis, and metabolomic analysis. The results showed several enzymes and cellular processes involved with the resistance and reduction capacity of hexavalent chromium, including quorum sensing. However, the authors believe that a single bacterial strain in this case is more efficient in bioremediation than communities because of the oxidative stress generated (Chai et al., 2019). The issue of multiple metabolic steps is a major issue here; therefore, a more detailed approach should be taken with communities subjected to chromium or other xenobiotic contamination.

Positive practical results are more likely to be achieved when the genetic and biochemical context of different species of bacteria is known in more depth, as has been shown for sulfamethoxazole-degrading strains, *Vibrio alginolyticus* and *Pseudomonas pseudoalcaligenes*, in the presence of bacterial communities with different ecological functions, as ammonia oxidation, photosynthesis, and nitrogen fixation, can restore the environmental balance and water quality in milkfish culture ponds (Chang et al., 2019).

The third generation of high-throughput DNA sequencing is based on platforms of true single molecular sequencing (tSMS) of Helicos Biosciences, the PacBio of Pacific Biosciences, and the nanopore single-molecule technology of Oxford Nanopore Technologies. Zhang et al. (2019) used the PacBio platform to obtain the sequence of complete genome of *Klebsiella pneumoniae* 2N3. These authors obtained insights into genes that encode degradation enzymes of sulfonylurea herbicides and support for further exploration of degradation pathways for possible use for bioremediation purposes. Using this technology, the authors were able to describe regulation systems for biodegradation, including esterase SulE and cytochrome P450.

Despite the knowledge of response systems to herbicides obtain through omics approaches and the possibilities of efficient bioremediation by bacterial communities, there is a possible pitfall for developing countries. Data from the Food and Agriculture Organization of the United Nations (http://www.fao.org/statistics/en/) showed that, in 2016, there was no proportional relationship between pesticide use and percentage of undernourished in continents. For example, Europe had the lowest rates of undernourished people in the world, with 1.5% prevalence of severe food insecurity, using only 1.66 kg/ha of pesticides. By contrast, Asia had the one of the highest malnourishment rates in the world (11.4%) but with high pesticide use rates (3.64 kg/ha). Despite the possibilities of manipulating bacterial communities for more sustainable bioremediation processes, the very concept of omics introduces several problems: they are more expensive and complex approaches than the traditional analyses and require more powerful bioinformatics systems to analyze the large amounts of data generated (Pathak et al., 2018). Without help to implement and fund omic technologies, developing agricultural countries will have greater problems in achieving self-sufficiency to solve problems of environment.

## 12 Conclusions

One of the guiding principles for sustainable use of herbicides in agriculture is that they should only target weed-specific systems such as photosynthesis-related enzymes, amino acid production, and growth regulators. Unfortunately, the improper use of herbicides results in increased waste in the environment, which may lead to the selection of herbicide-resistant weeds and decreased viability of non-target organisms, including soil and water microbial communities.

Several strategies are used to mitigate this situation. One of them is bioremediation, based on the enzymatic capacity of microorganisms responsible for herbicide degradation, transformation, or mineralization. There are limitations to this approach, including the production of more toxic metabolites via incomplete herbicide degradation processes. Herbicides cause oxidative stress; therefore, for degradation processes to occur, microorganisms need more plastic antioxidant mechanisms.

The various techniques for mitigating herbicides in the environment have low efficiencies in elimination of waste, generating important environmental liabilities. Alternatives based on mixed microbial communities that showing higher genetic and metabolic diversity appear to be more efficient than single strains in bioremediation. These communities present higher levels of gene complexity and interactions of several metabolic pathways, quorum sensing communication, and organization of microbial populations in biofilms, all of which requires molecular approaches (the omics) to obtain deeper access to the large amounts of generated data. Bioremediation processes based on integrated bacterial consortia and manipulated by quorum sensing may represent the paradigm shift needed to achieve herbicide mineralization in a more efficient and sustainable manner than currently occurs. Nevertheless, it is necessary that developing countries, which are major food producers and consumers of pesticides, have access to these techniques so as to achieve sustainable production.

## Declarations

### Author contribution statement

All authors listed have significantly contributed to the development and the writing of this article.

### Funding statement

This work was supported by the Coordination for the Improvement of Higher-Level Personnel (10.13039/501100002322CAPES), the National Council of Technological and Scientific Development (10.13039/501100003593CNPq), and the Foundation for Research Support of the State of Paraná (Fundação Araucária).

### Data availability statement

Data included in article.

### Competing interest statement

The authors declare no conflict of interest.

### Additional information

No additional information is available for this paper.

## Appendix A Supplementary data

Supplementary Material Table 1_V2.docx Supplementary Material Table 1_V2.docx
