# Supplementary material for: Herbicide bioremediation: from strains to bacterial communities
Source: Heliyon. 2020 Dec 24;6(12):e05767. doi: 10.1016/j.heliyon.2020.e05767 (PMC7773584; doi:10.1016/j.heliyon.2020.e05767)
Supplement: Supplementary Material Table 1_V2.docx [file mmc1.docx]

Table 1: Herbicides discussed and their molecular structures showing electronegative residues, their mode of action, and examples of degrading bacteria.

| Herbicides | Molecular Structure | Mode of Action | Chemical family | HRAC Classification | Degrading Bacteria | Reference |
| --- | --- | --- | --- | --- | --- | --- |
| Glyphosate | 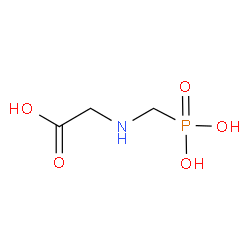 | Inhibition of 5-enolpyruvylshikimate-3-phosphate synthase | Glycine | G | *Escherichia coli* | Jin et al., 2007 |
| 2,4-D | 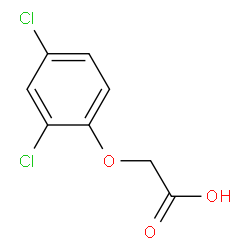 | Synthetic auxin | Phenoxy-carboxylic acid | O | *Acinetobacter* sp*., Serratia marcescens, Stenothrophomonas maltophilia, Flavobacterium* sp*. and Penicillium* sp*.* | Silva et al., 2007 |
| Quinclorac | 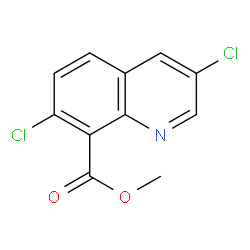 | Synthetic auxin | Quinolone carboxylic | O | *Bacillus megaterium* | Liu et al., 2014 |
| Thiobencarb | 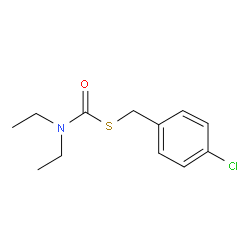 | Inhibition of lipid synthesis | Thiocarbamate | N | *Acidovorax* sp*.* | Chu et al., 2017 |
| Mesotrione | 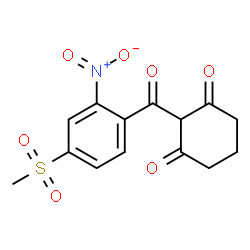 | Inhibition of 4-hydroxyphenyl-pyruvate-deoxygenase | Triketone | F2 | *Pantoea* *ananatis,*  *E. coli,*  *Bacillus* *megaterium* | Olchanheski et al., 2014;  Pileggi et al., 2012;  Prione et al., 2016 |
| Atrazine | 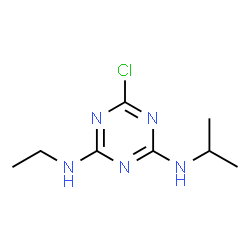 | Inhibition of photosynthesis at Photo System II | Triazine | C1 | *Pseudomonas* ADP and *Arthrobacter aurescens* | Mongodin et al., 2006;  Sadowsky et al., 1998;  Seffernick et al., 2007 |
